# Supplementary material for: Fatty acid composition, total phenolic and total flavonoid contents, and antioxidant activity of Niger seed (Guizotia abyssinica) accessions collected from major producer areas of Ethiopia
Source: PLoS One. 2025 Jan 13;20(1):e0317397. doi: 10.1371/journal.pone.0317397 (PMC11729926; doi:10.1371/journal.pone.0317397)
Supplement: Attachment — Submitted filename: Response to Reviewers.docx [file pone.0317397.s001.docx]

September 15, 2024

Dr. Marie Yayinie

Debre Tabor University, Ethiopia

Email: *marieyayinie@gmail.com*

Note to the reviewers of the manuscript: "**Fatty acid composition, total phenolic and total flavonoid contents, and antioxidant activity of Niger Seed (Guizotia Abyssinica) accessions collected from major producer areas of Ethiopia.**

Ref: Submission ID: PONE-D-24-16831

**Dear Sir/Madame**

**Editor in chief of the PLOS ONE Journal**

We would like to kindly thank you for considering our submission, as well as the reviewers who carefully analyzed our paper, helping us to improve the presentation of our work. As requested, our manuscript was extensively revised, according to reviewers as well as editors’ comments’.

Please find below our detailed responses to reviewers' comments, as well as the editor’s view outlining the changes made in the revised manuscript, as suggested. All the changes in the manuscript were highlighted in red font.

Note concerning the reviewer and editor’s comments:

Editor’s Comment

1. Please ensure that your manuscript meets PLOS ONE's style requirements, including those for file naming

Response: We have now made intensive efforts to make our manuscript meet your journal's requirements and named files as you suggest.

2. We suggest you thoroughly copy edit your manuscript for language usage, spelling, and grammar. If you do not know anyone who can help you do this, you may wish to consider employing a professional scientific editing service

Response: We thoroughly reviewed the manuscript and edited the language, and tried to minimize errors related to grammar and spelling.

3. In your Methods section, please provide additional information regarding the permits you obtained for the work. Please ensure you have included the full name of the authority that approved the field site access and, if no permits were required, a brief statement explaining why.

Response: We appreciate the editor allowing us to clarify our methods concerning permits. This study is part of an M.Sc. thesis, and the Graduate Program Office of Dilla University approved our research proposal. Basically, the university is responsible for issuing ethical clearance for issues requiring permits before the proposal is approved for funding. Fortunately, our study did not demand the issuing permit letter from the university’s ethical board office. Therefore, aside from the approval from Dilla University's Graduate Office, we did not obtain any additional permit letters. It is important to note that Dilla University is a public institution under government oversight, which ensures compliance with relevant legal requirements.

4. We note that your Data Availability Statement is currently as follows: All relevant data are within the manuscript and its Supporting Information files.

Response: We have revised the data availability statement as follows. Data is available on request from the corresponding author.

**Reviewer #1**

- This paper addresses the extraction method, compositions, and antioxidant activity assessment of Niger Seed in different regions and zones of Ethiopia. However, there is a lack of detailed exploration of the correlation between the compositions of Niger seed and their antioxidant potential, as well as their prospective applications. Furthermore, the paper falls short of providing adequate supporting evidence for the significant claims put forth

**Response:** We accept it and we have significantly revised our manuscript incorporating the comments given particularly at the introduction, and result and discussion part.

- The author is advised to include additional content in the paper, such as providing more background information on topics like saturated and unsaturated fatty acids, exploring Niger seed applications as inactive ingredients, and correlating their composition can be applied in various industrial sectors i.e pharmaceuticals, hygiene care, and food.

**Response:** We fully accept this comment and incorporated the detailed discussion on the saturated and unsaturated fatty acids present in the samples in the revised manuscript.

- Furthermore, it is suggested that the authors incorporate insights into the differences in localities, climates, locations, and soil types among regions and zones to better elaborate on the relationship between Niger seed composition and specific geographical factors. Expand the discussion from the results obtained and consider reporting the calibration curve as supplementary data rather than part of the main results.

**Response:** We fully accepted the comments and addressed them in the revised manuscript.

- Recommended to address the ethics statement and approval regarding field sampling permits

**Response:** We appreciate the editor allowing us to clarify our methods concerning permits. This study is part of an M.Sc. thesis, and the Graduate Program Office of Dilla University approved our research proposal. Basically, the university is responsible for issuing ethical clearance for issues requiring permits before the proposal is approved for funding. Fortunately, our study did not demand the issuing permit letter from the university’s ethical board office. Therefore, aside from the approval from Dilla University's Graduate Office, we did not obtain any additional permit letters. It is important to note that Dilla University is a public institution under government oversight, which ensures compliance with relevant legal requirements

- The distinction between 80% and 100% methanol extraction is noted in the scavenging antioxidant, total phenolic content, and total flavonoid content but not in the fatty acid compositions, prompting a query about potential differences in this aspect.

**Response:** Since we have adopted the well-known extraction method, ,i.e., the Folch method, to extract fatty acids. Secondly, fats are usually soluble in non-aqueous media, and we have not yet tried to extract them using aqueous methanol. Hence a mixture of methanol and chloroform was used for extraction purposes as recommended by FolCh et al

- Additionally, the study mentions the poor correlation of flavonoids and phenolics contents with antioxidant activity, suggesting the need to support this observation with additional scientific reports to support the findings.

**Response:** The presence and absence of a strong correlation between the parameters studied have been discussed in detail, and supporting evidence has been given for each assertion we made in the revised manuscript.

- There is a lack of reporting on the statistical analysis in Tables 4 and 5, necessitating a careful interpretation of each superscript (a-d) from the Tukey test analysis to clarify the comparators for the readers. A detailed explanation of the statistical findings is essential to enhance the understanding of the results presented in the tables.

**Response:** Thank you for pointing out these drawbacks. It has now been further interpreted accordingly under each table in addition to the information given in the caption. The tables number are now changed to Table 3 and 4 as table 2 is removed based on your comment

- Based on these preliminary findings, the authors are encouraged to outline future research that can stem from the analysis and discuss the potential implications.

**Response:** We have included the implications of our research findings and also outlined future research in the conclusion part of the manuscript.

- Table 2 does not directly align with critical aspects or does not contribute significantly to the understanding of the composition of Niger Seed; it may be deemed irrelevant in the context of this paper.

**Response:** Thank you for the recommendation. We accept it and remove this table from the revised manuscript by pasting the data (total concentration at the bottom of the next table (table 3) which is renamed as table 2 in the revised manuscript.

- There have been identified typographical, spelling, and grammatical errors that require careful attention. For instance, on Page 8, the term "IC50%", representing the inhibitory concentration, should not be presented as a percentage since it pertains to concentration or dosage. On Page 6, the phrase "sum upping" would be more appropriately replaced with "summing up."

**Response:** We have thoroughly edited the manuscript and removed, if not minimized, errors related to typos, grammar, etc.

- Page 4; Materials and methods (Na_2_WO_4_.2H_2_O) is Sodium tungstate dehydrate.

**Response:** Thank you**.** We accept and correct it in the revised manuscript as Sodium tungstate dihydrate

**Reviewer #2**:

- The original and interesting aspect to this work relates to sampling from different geographical/climatic regions, however the authors fail to make a strong case for this. The stated "core target" of this study was the assessment of the nutritional components of niger seed (Guizotia abyssinica) based on geographical origin, cultivation season and seed variety however the authors didn't establish how they intended to do address each of these questions nor how their findings related to their stated objectives. A more logical introduction setting out your objectives, structured study design including a map of sampling locations to assist the reader would have been helpful.

**Response:** We made rigorous revisions to the manuscript, and all the issues raised by our esteemed reviewer are logical, and we have addressed all of them. We have also included a map showing the sampling zones and regions.

- What seed varieties were sampled at each sampling site? Could differences in variety or seasonal variations account for your observations rather than geographical location, without this information we cannot draw any firm conclusions from your findings.

**Response:** We have included information on the seed variant collected for this study. Details can be seen in the section “Sample collection”.

- Data from one of their five sampling sites (HG/Wolega) falls well outside the expected range for fatty acid content in Guizotia abyssinica based on previously published findings and this discrepancy has not been be accounted for.

**Response**: We have significantly revised the manuscript and provided possible reasons for the high and low values of the parameters studied in each sample. We have also included literature support for each assertion we made.

- The Ferric Ion Reducing Antioxidant Power (FRAP) is a more reliable assay for assessment of antioxidant activity and shouldn’t be too difficult to establish in your lab.

**Response:** There are indeed several methods of assessing the antioxidant activity of plant extracts, including the FRAP assay. However, most of the reported literatures recommend the DPPH assay method because of many factors, such as its easy, fast, and low-cost method. Hence we decided to focus on the DPPH assay method.

Silva, F., Veiga, F., Cardoso, C., Dias, F., Cerqueira, F., Medeiros, R. and Paiva-Santos, A.C., 2024. A rapid and simplified DPPH assay for analysis of antioxidant interactions in binary combinations. *Microchemical Journal*, 202, p110801. *https://www.mdpi.com/2227-9717/11/8/2248*

- The manuscript would have benefited from professional editing prior to submission.

**Response:** We have significantly edited the manuscript with respect to its language and content

- I have been unable to find any supporting information files despite the authors stating they are included in the submission.

**Response:** We have included relevant data in the manuscript. We have no supporting information.

Reviewer #3:

- You have to track the mechanism of action using suitable computational techniques to support your data, such as network pharmacology, molecular dynamic simulation. More biological assays are required comparable studies with other species could be useful for data support

**Response:** We respect our reviewer’s comments. However, our intention was to quantify the fatty acid content and total flavonoid content of the five niger seed samples and correlate their composition with the region of origin as well as correlate the phytochemical composition with the antioxidant activity of the seed extract. We wish to link the antioxidant activity data with some biological assay, but such a study is beyond our scope.
