# Supplementary material for: Fatty acid composition, total phenolic and total flavonoid contents, and antioxidant activity of Niger seed (Guizotia abyssinica) accessions collected from major producer areas of Ethiopia
Source: PLoS One. 2025 Jan 13;20(1):e0317397. doi: 10.1371/journal.pone.0317397 (PMC11729926; doi:10.1371/journal.pone.0317397)
Supplement: Attachment — Submitted filename: Response to Reviewers.docx [file pone.0317397.s002.docx]

December 16, 2024

Dr. Marie Yayinie

Debre Tabor University, Ethiopia

Email: [*marieyayinie@gmail.com*](mailto:marieyayinie@gmail.com)

Note to the reviewers of the manuscript: "**Fatty acid composition, total phenolic and total flavonoid contents, and antioxidant activity of Niger Seed (Guizotia Abyssinica) accessions collected from major producer areas of Ethiopia.**

Ref: Submission ID: PONE-D-24-16831R1

**Dear Sir/Madame**

**Editor in chief of the PLOS ONE Journal**

We would like to kindly thank you for considering our submission, as well as the reviewers who carefully analyzed our paper, helping us to improve the presentation of our work. As requested, our manuscript was extensively revised, according to reviewers as well as editors ‘comments’.

Please find below our detailed responses to reviewers' comments, as well as editor’s view outlining the changes made in the revised manuscript, as suggested. All the changes in the Revised manuscript with Track change were highlighted in red font.

Reviewer #4:

Comment 1:. The paper still contains some spelling error. Please thoroughly check every words.

Response: We have significantly edited the manuscript to minimize errors related to grammar

Commnet 2: . I think it would be better if you provided separate tables for saturated and unsaturated fatty acid profiles or a single table with clear mentions of total saturated and total unsaturated fatty acid content to highlight the importance of Niger Seeds in nutritional point of view.

Response: We have included the total saturated and unsaturated fatty acids in Table 2 bottom part. We have also included the associated discussion on page 11

Reviewer #5:

The paper presents a good comparative study of four main characteristics of Niger seed oil.
The manuscript already underwent thorough revision and most of the comments held against the original version have been solved already.
However, several comments and advices to the authors still can be offered:

1) The content and ratio of fatty acids, phenolics and antioxidants in seeds and seed oil is often different from those in the oil, as known ie. for Moringa oleifera <https://doi.org/10.1016/j.sajb.2023.02.010> or even depend on oil production method. Was there an attempt to analyse also oil made from the samples? Were the results comparable? How about the effect of oil preparation method?

Response: We have not analyzed the phenolic content of the oil. Rather we have done on the phenolic content from the seed extract. It is true the phenolic contet of the oil fluctuate based on the oil processing procedure.

2) In the age of green solvents and sustainable sources, methanol-based solvent system is recommended for the extraction. While, no doubt, it is efficient and relatively low-cost, the method will be hard to apply for routine quality monitoring in most of modern countries due to environmental issues? Was there an attempt to use more environmentally friendly extractants, but the yield was too low? Did you try to optimize the conditions for another solvent? If so, please add the information for the benefit of others.

Response: As our respected reviewer noted, methanol and aqueous methanol are good solvents for the extraction of plant phytochemicals. Since these solvents are cheap and easily accessible, we have not yet investigated different techniques to extract the phenolic chemicals. Our future work may consider looking at different ways. Hence we thank our review’s suggestion.
